# Grasping and cutting points detection method for the harvesting of dome-type planted pumpkin using transformer network-based instance segmentation architecture

**DOI:** 10.3389/fpls.2023.1063996

**Published:** 2023-04-18

**Authors:** Jin Yan, Yong Liu, Deshuai Zheng, Tao Xue

**Affiliations:** School of Computer Science and Engineering, Nanjing University of Science and Technology, Nanjing, China

**Keywords:** keypoint detection, stem instance segmentation, transformer, point rendering, pumpkin harvesting

## Abstract

An accurate and robust keypoint detection method is vital for autonomous harvesting systems. This paper proposed a dome-type planted pumpkin autonomous harvesting framework with keypoint (grasping and cutting points) detection method using instance segmentation architecture. To address the overlapping problem in agricultural environment and improve the segmenting precision, we proposed a pumpkin fruit and stem instance segmentation architecture by fusing transformer and point rendering. A transformer network is utilized as the architecture backbone to achieve a higher segmentation precision and point rendering is applied so that finer masks can be acquired especially at the boundary of overlapping areas. In addition, our keypoint detection algorithm can model the relationships among the fruit and stem instances as well as estimate grasping and cutting keypoints. To validate the effectiveness of our method, we created a pumpkin image dataset with manually annotated labels. Based on the dataset, we have carried out plenty of experiments on instance segmentation and keypoint detection. Pumpkin fruit and stem instance segmentation results show that the proposed method reaches the mask mAP of 70.8% and box mAP of 72.0%, which brings 4.9% and 2.5% gains over the state-of-the-art instance segmentation methods such as Cascade Mask R-CNN. Ablation study proves the effectiveness of each improved module in the instance segmentation architecture. Keypoint estimation results indicate that our method has a promising application prospect in fruit picking tasks.

## 1 Introduction

Agriculture is the foundation of people’s livelihood. To effectively harvest crops, fruits and vegetables, researchers have made efforts from different aspects, for instance, nutrient supply (Sun et al., 2022), disease prevention (Yang et al., 2022), postharvest preservation (Pan et al., 2023) and so on. Crop, fruit and vegetable picking is often the most labor-intensive part of the entire production chain. Therefore, intelligent picking robots have become a research hotspot. Among them, accurate detection is a prerequisite for intelligent picking, and many vision-based fruit and vegetable detection works have been launched.

In recent years, deep learning applications have attracted great attention and made great breakthroughs in image processing tasks (Liu et al., 2021a; Bhatti et al., 2023), the research on learning-based fruit and vegetable detection also moves forward. Liu et al. (2019a) trained a Support Vector Machine (SVM) classifier utilizing the Histograms of Oriented Gradients (HOG) descriptor to detect mature tomatoes. The proposed machine learning method’s recall, precision, and F1 scores are 90.00%, 94.41%, and 92.15%, respectively. Sun et al. (2019) designed a GrabCut model based on the visual attention mechanism for fruit region extraction, then applied the Ncut algorithm to segment the extracted fruits. The recognition method achieves the F1 score of 94.12% and an error of 7.37%. Deep learning (DL) has developed rapidly in these years, and because of its excellent performance, DL has been applied in many fields, including agriculture. Yuan et al. (2020) applied SSD to detect tomatoes in the greenhouse with the backbone of Inception V2, and the network achieves an average precision of 98.85%. Bresilla et al. (2019) set up a fruit detection network based on YOLO. The network can be trained to detect apples and pears without classifying them. The architecture shows an accuracy of more than 90% fruit detection. Fu et al. (2020) compared two Faster R-CNN based architectures ZFNet and VGG16, employed to detect apples in images. The results indicate that the network with VGG16 achieves the highest average precision (AP) of 0.893.

It can be seen that the accuracy and speed of fruit and vegetable detection can meet the requirements of practical applications. However, deep learning-based detection frameworks only generate coarse boundaries, and many pixels irrelevant to the detected fruit or vegetable are also included in the bounding box. In order to obtain more abundant information, some scholars have carried out researches on fruit or vegetable instance segmentation. Instance segmentation combines the advantages of semantic segmentation and object detection and identifies each object instance of each pixel for every known object within an image. With the help of instance segmentation, fruits or vegetables can be assigned to different instances with pixel accuracy.

Ganesh et al. (2019) presented a deep learning approach, named Deep Orange, to detect and pixel-wise segment oranges based on Mask R-CNN. Gonzalez et al. (2019) proposed a network based on Mask R-CNN for blueberry detection and instance segmentation. The authors tested the performances of several backbones such as ResNet101, ResNet50, and MobileNetV1. Jia et al. (2020) improved Mask R-CNN through the fact as the feature extraction, RoI acquisition, and mask generation so that the network is more suitable for recognizing and segmenting overlapped apples. Also based on the well-known Mask R-CNN network, Perez-Borrero et al. (2020) designed a new backbone and mask network, removed the object classifier and the bounding-box regressor and replaced the non-maximum suppression algorithm with a new region grouping and filtering algorithm to better segment instances of strawberry. The same research team (Perez-Borrero et al., 2021) proposed another strawberry instance segmentation methodology based on the use of a fully convolutional neural network. Instance segmentation is achieved by adding two new channels to the network output so that each strawberry pixel predicts the centroid of its strawberry. The final segmentation of each strawberry is obtained by applying a grouping and filtering algorithm. Liu et al. (2019b) improved Mask R-CNN to detect and segment cucumbers by designing a logical green operator to filter non-green backgrounds and adjusting the scales and aspect ratios of anchor boxes to fit the size and shape of cucumbers.

In the actual picking applications, the key operating points are often generated in the fruit stem area, so the detection of stems should be taken seriously. Some scholars have focused their attention on fruit stem detection.

Sa et al. (2017) made use of an RGB-D sensor to acquire color and geometry information and utilized a supervised-learning approach for the peduncle detection task. Yoshida et al. (2018) used the support vector machine to classify the point cloud data, clustering to obtain fruit stem pixels, and then looking for cutting points. Luo et al. (2018) studied the detection of cutting points on stems of overlapping grape clusters. After segmenting individual clusters using machine learning method, a geometric constraint method is then used to determine the cutting point in the region of interest of each cluster’s stem. Sun et al. (2021) developed a deep learning-based top-down framework to detect keypoint on the bearing branch, enabling branch pruning during fruit picking. This work only detects citrus branch keypoint without segmentation. Kalampokas et al. (2021) applied a regression convolutional neural network (RegCNN) for executing a stem segmentation task and determined the cutting point on the stem based on a geometric model. Chen et al. (2021) proposed a banana stalk segmentation method based on a lightweight multi-feature fusion deep neural network. The methods in both (Kalampokas et al., 2021) and (Chen et al., 2021) can only segment the stem of a single cluster of grape or banana. Wan et al. (2022) proposed a real-time branch detection and reconstruction method applied to fruit harvesting. To segment the branches separately, the authors first detect branch region boxes using YOLOv4, then utilize image segmentation to locate the branch boundaries. Next, the division of precise boxes belonging to the same branch is achieved based on the branch growth trend constraints. Rong et al. (2021) proposed a method to localize the peduncle cutting point and estimate the cutting pose. The authors first detect tomatoes *via* YOLOv4 and then segment fruit and peduncle masks by YOLACT++. The segmented peduncle mask is fitted to the curve using least squares and three key points on the curve are found. Chen and Chen (2020) proposed a methodology to identify the plucking points of tea shoots using machine vision and deep learning. The authors first localize the one tip with two leaves regions through Faster-RCNN, then identify the plucking areas using FCN. The plucking point is determined as the centroid of the plucking area. The approaches in (Wan et al., 2022) (Rong et al., 2021), and (Chen and Chen, 2020) treat detection and stem instance segmentation as two separate networks.

**Figure 1:**
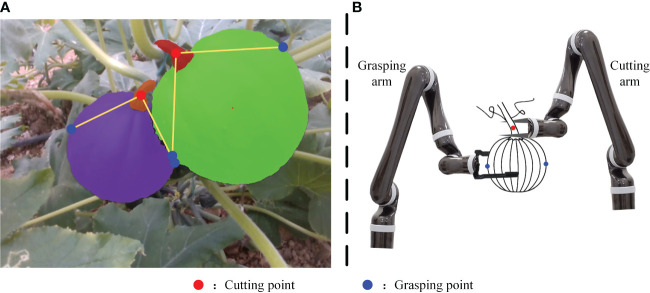
Example of pumpkin keypoint detection framework output **(A)** and pumpkin picking illustration **(B)**.

As a nutritious crop, there are few studies on pumpkin detection. Wittstruck et al. (2020) and Midtiby and Pastucha (2022) have conducted researches on large-scale pumpkin yield estimation. The datasets are captured by UAVs from the air. To the best of the authors’ knowledge, there is currently no dataset consisting of close-range pumpkin images and devoted to autonomous pumpkin harvesting. In this paper, we established a dataset on two varieties of pumpkin, and the instance masks of pumpkin fruit and pumpkin stem are labeled manually. The pumpkin stem is thick and it is hard to tear off or twist off the pumpkin fruit with one end effector. As is illustrated in **Figure 1B**, an ideal way to pick the pumpkin is utilizing two arms or one arm with two end effectors, one to grasp and another to cut. The detection of pumpkin stems cannot be ignored during automatic picking. This paper presents a pumpkin autonomous picking framework with keypoint detection and instance segmentation method. Firstly, pumpkin fruit and stem masks can be generated by instance segmentation method as shown in **Figure 1A**. Then, through the keypoint detection algorithm, relationships among the fruit and stem instances are determined and keypoints are localized as marked in **Figure 1**, where red points are cutting points, blue points are grasping points, and yellow lines link one stem and one fruit that belong to one pumpkin instance. Main contributions of our work are three folds:

1) We propose a novel pumpkin autonomous picking framework with grasping and cutting point detection method using instance segmentation architecture. The keypoint detection algorithm can model the relationships among the fruit and stem instances as well as estimate grasping and cutting keypoints.
2) This paper presents a pumpkin fruit and stem instance segmentation architecture based on deep learning and applying a transformer backbone and point rendering mask head. Compared with several state-of-the-art instance segmentation methods, the proposed method shows significant performance advantages in both metric evaluation and visualization analysis.
3) To validate the effectiveness of our method, we created a pumpkin image dataset with manually annotated labels. Downstream tasks such as image classification, pumpkin detection and instance segmentation can be deployed on the database.

The remainder of this paper is arranged as follows. Section 2 introduces the dataset and our method. Section 3 presents the results and analyses. Finally, conclusions are summarized in Section 4.

## 2 Materials and methods

In this paper, we perform instance segmentation on pumpkin fruit and stem. Then, we detect and localize the grasping points and cutting point using the proposed keypoint detection algorithm. To complete this research, we first collect pumpkin images to establish the dataset.

### 2.1 Data acquisition

**Figure 2:**
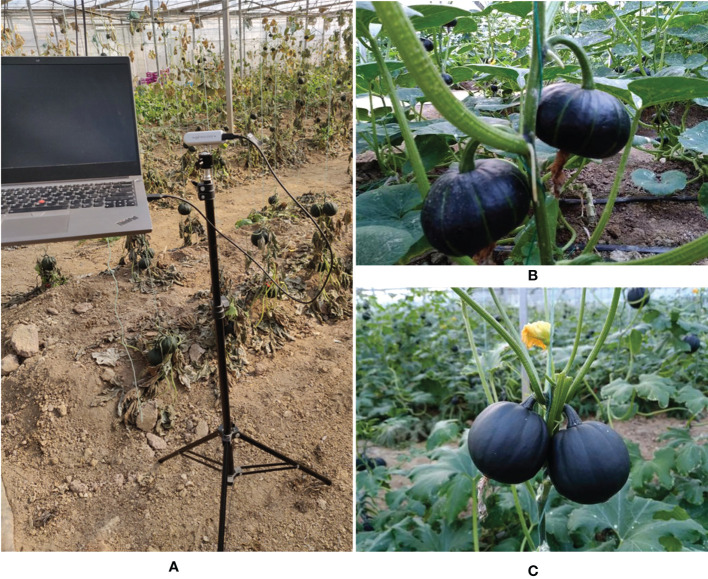
Image collection environment and pumpkin images. **(A)** Image capture scene. **(B)** Original Bebe pumpkin image. **(C)** Original Hazel pumpkin image.

This paper establishes a pumpkin dataset containing two varieties of pumpkin (Bebe pumpkin and Hazel pumpkin). The dataset was collected in Tangshancuigu modern agriculture demonstration zone, Nanjing, China. We used three different capture devices (Intel RealSense D435i, One Plus 6T smartphone, and Apple iPhone 13 Pro smartphone) to collect a total of 679 ripe pumpkin images. The original image pixels are 1280×720, 4608×3456, and 4032×3024, respectively. To better train the images, we resized the high-resolution images from 4608×3456 and 4032×3024 to 640×480. The resolutions of final images in the dataset are 1280×720 and 640×480. The dataset collection environment and real image examples are shown in **Figure 2**.

**Figure 3:**
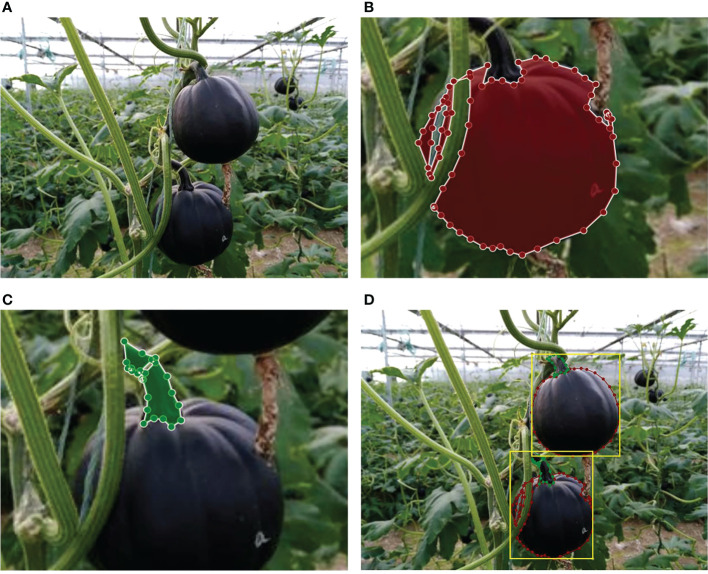
Illustration of the image annotation process. **(A)** Original image. **(B)** Polygonal annotation and extraction of the pumpkin fruit mask. **(C)** Polygonal annotation and extraction of the pumpkin stem mask. **(D)** Annotated images, red polygons are pumpkin fruits, green polygons are pumpkin stems, yellow boxes are pumpkins.

Through the review above, the pixel-wise segmentation of fruits and the labeling of fruit stems are very important. Therefore, we manually annotated the pixel-level instances of the pumpkin fruit and stem, as well as the pumpkin box containing one fruit and stem (see **Figure 3**). The labeling software we used is Labelme. **Table 1** shows the distribution of the dataset.

**Table 1:** Distribution of the dataset.

	Images	Fruit instances	Stem instances	Pumpkin bounding boxes
Bebe	354	608	552	608
Hazel	325	676	516	676
Total	679	1284	1068	1284

**Figure 4:**
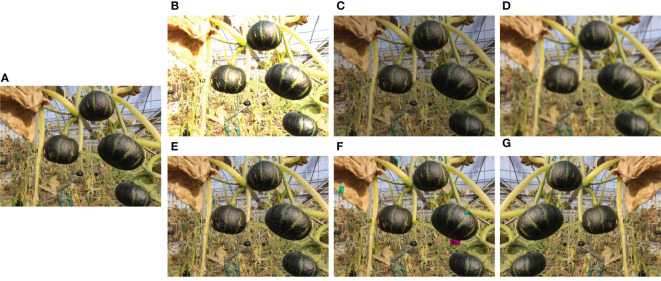
Data augmentation. **(A)** Original image; **(B)** Brightness enhancement; **(C)** Brightness reduction; **(D)** Gaussian blur; **(E)** Noise; **(F)** Cutout; **(G)** horizontal flip.

The data is split into a training set and a testing set with a ratio of 80:20, where 543 images belong to training set and 136 images are in the testing set. Differing from the general structured scene, agricultural environment is a typical unstructured scene. The key problems faced during image collection in agricultural environment are large changes in illumination, a lot of dust, and frequent overlaps of fruit branches and leaves. To simulate the agriculture environment and enhance the generalization and robustness of deep neural network, we augment the dataset by changing brightness, blurring the image, adding noise, and cutout operation as shown in **Figure 4**. In addition, the horizontal flip is operated with a probability of 0.5 during training. After data augmentation, the training set contains 3258 images.

### 2.2 Pumpkin fruit and stem instance segmentation

**Figure 5:**
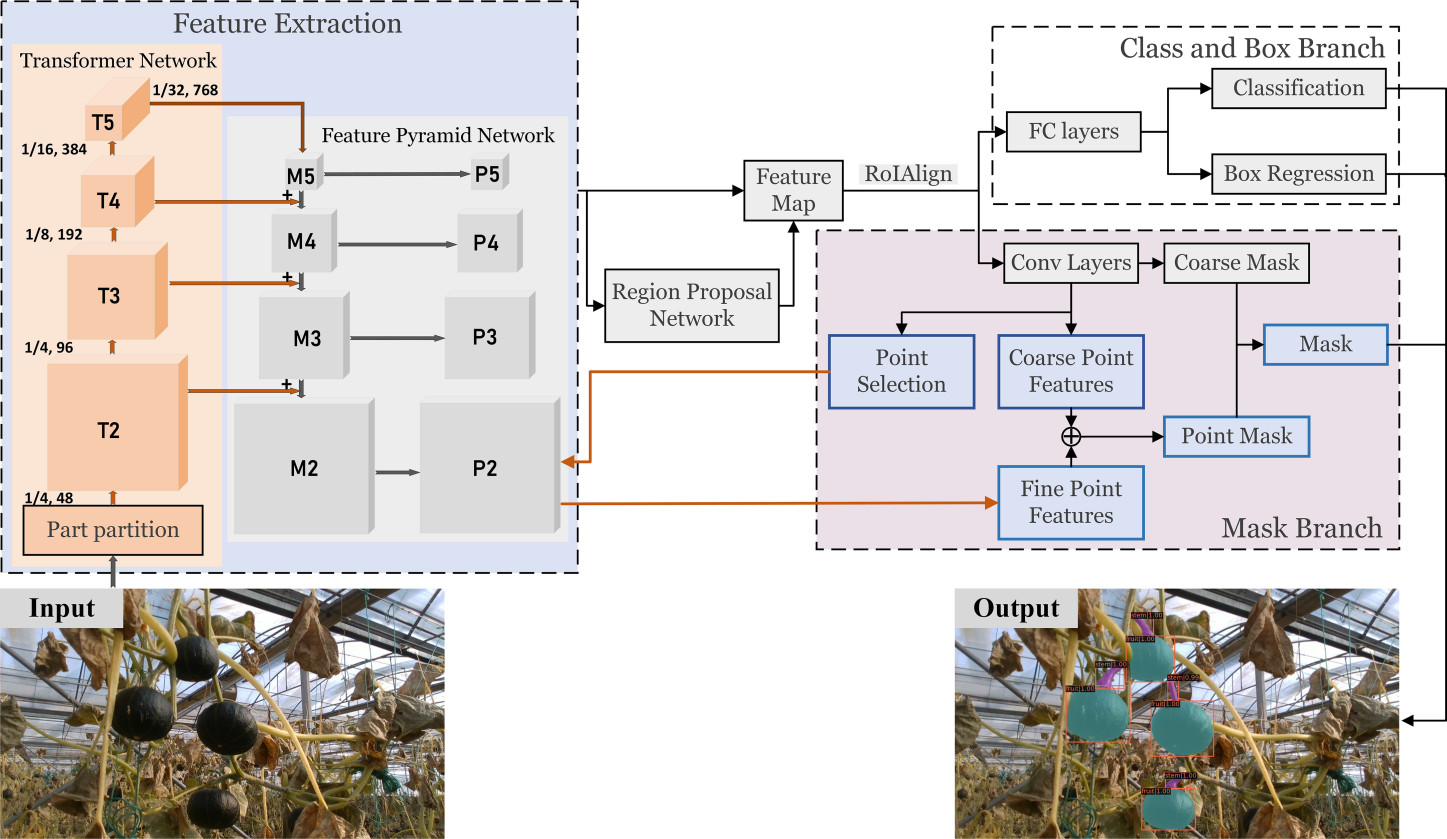
Pumpkin fruit and stem instance segmentation framework.

The agricultural environment is a typical unstructured environment with complex background. Due to ‘free growth’ and overlapped fruits, stems, branches, and leaves, fine instance segmentation in fruit harvesting environment becomes a challenging work. In this paper, we proposed a pumpkin fruit and stem instance segmentation framework as illustrated in **Figure 5**. The main feature of this framework is introducing a transformer network to replace the commonly used convolutional neural network (CNN). The transformer network helps effectively extract image features, improve instance segmentation accuracy, and reduce model computational complexity. In addition, to deal with the overlapping phenomenon that often occurs in the harvesting environment, we add a hard point selection module to the mask branch. Coarse features are concatenated with fine features from the output of the feature pyramid network (FPN) to classify those hard points and then generate the final fine mask.

Compared with the literature in the previous review, our framework achieves the end-to-end fruit and stem instance segmentation. The specific implementation is as follows. First, we introduce the Swin Transformer (Liu et al., 2021b) to the task of pumpkin fruit and stem instance segmentation and replace CNN (such as ResNet) to extract features. The feature extraction structure of this transformer network combined with a feature pyramid network (FPN) (Lin et al. (2017)). Hierarchical transformer and FPN are applied to generate a pyramid of feature maps with different sizes of a fixed number of channels (set to 256). Specially, we use four levels of feature maps denoted as {*P*2,*P*3,*P*4,*P*5}. *P*2, *P*3, *P*4 and *P*5 are generated by four transformer feature maps *T*2, *T*3, *T*4 and *T*5, convolving with 1×1 kernel *via* top-down connection mechanism. As a result, *P*2, *P*3, *P*4 and *P*5 have strides 4, 8, 16 and 32 respectively. Then a region proposal network (RPN) (Ren et al., 2015) is deployed to generate the feature map with anchors. *Via* RoIAlign (He et al., 2017) operation, fixed-size feature maps can be obtained. After fully connected (FC) layers, prediction results of the bounding box and classification are output. In a general way, fixed-size feature maps can generate mask predictions after several convolution operations. However, since the fixed size of the feature map is generally 7*7, it is tough to generate an accurate mask, especially at the fruit boundary. Therefore, we select the hard points in the edge area and combine the coarse features from the fixed-size feature map and the fine features from high-resolution feature map output by FPN to generate more refined point-wise label predictions. Details of transformer network and mask branch will be introduced in subsections.

#### 2.2.1 Transformer network

**Figure 6:**
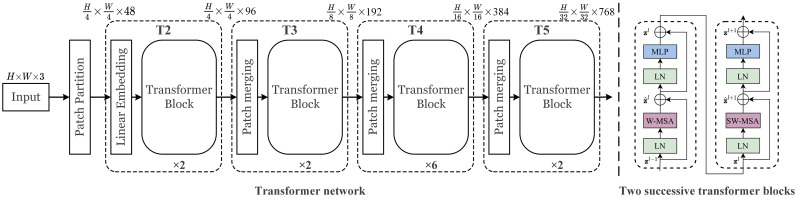
The architecture of transformer network.

Transformer has a great impact on the field of natural language processing (NLP) before. The proposal of vision transformer (ViT) (Dosovitskiy et al., 2020) breaks the gap between NLP and vision, and replaces the convolutional neural network with a pure transformation module to perform image classification tasks. Liu et al. (2021b) proposed a new visual transformer, called Swin Transformer, whose multi-scale and computationally inexpensive properties make it compatible with a wide range of vision tasks (image classification, object detection, semantic segmentation, etc.). An overview of the transformer architecture and transformer blocks we applied are presented in **Figure 6**. It first splits an input RGB image into non-overlapping patches (raw-valued features) by a patch partition operation. Then a linear embedding layer is applied to the raw-valued features to project them to an arbitrary dimension (set to 96). Several transformer blocks are applied to these patch tokens. To produce a hierarchical representation, the number of tokens is reduced by patch merging layers as the network gets deeper. Specific implementations are demonstrated in (Liu et al., 2021b).

#### 2.2.2 Mask branch

**Figure 7:**
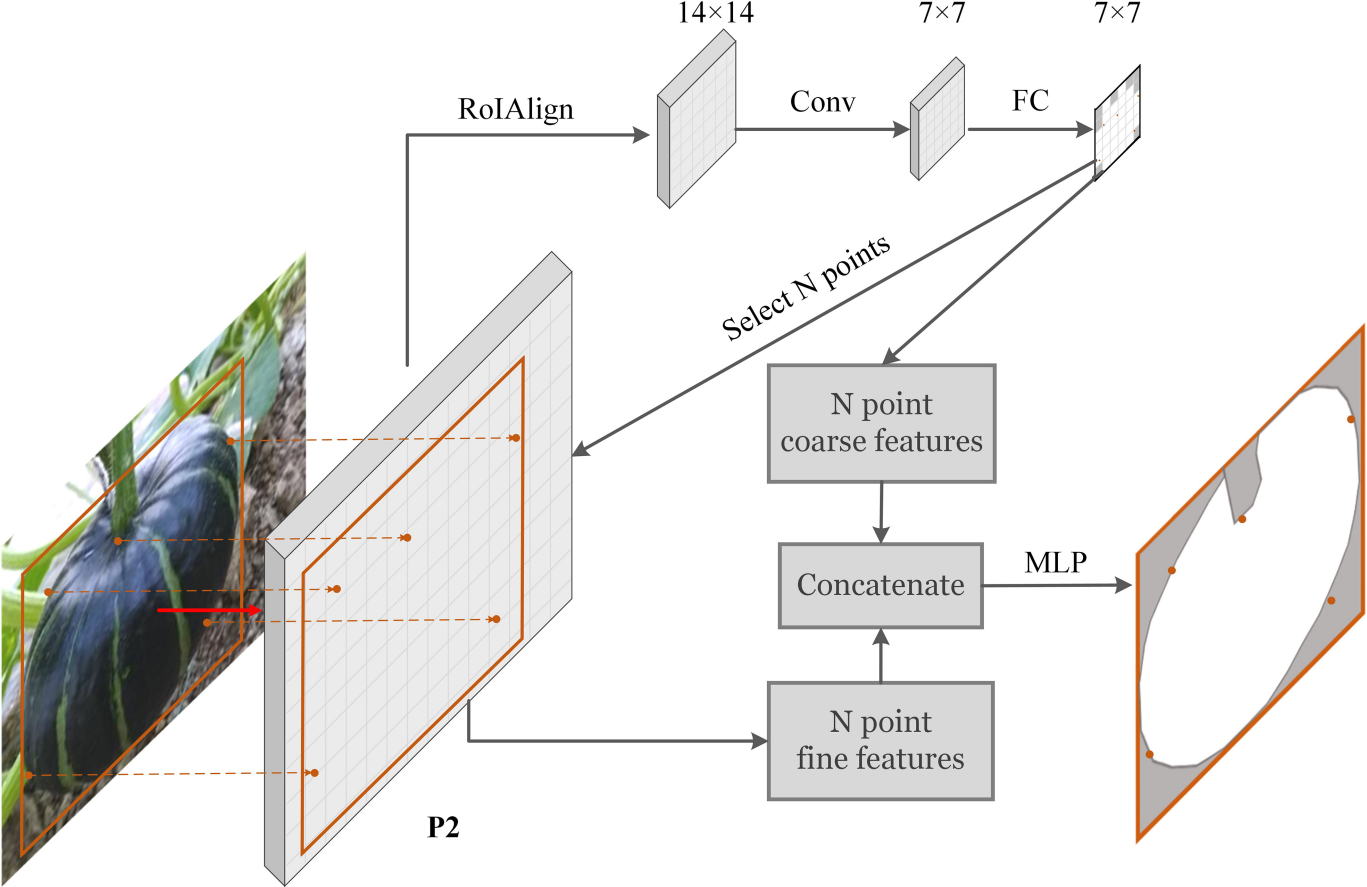
Scheme of point rendering mask head.

In the instance segmentation task of agricultural environment, due to the large-scale overlapping problem, fine segmentation of the target edges and overlapping edges is challenging. Research in (Li et al. 2017) shows that in the segmentation task, most of the hard pixels (about 70%) are at the edge of the object. Point rendering method (Kirillov et al., 2020) we applied is devoted to segmenting these blurry pixels finely. **Figure 7** depicts the main idea of point rendering. Point rendering includes three steps:

##### 2.2.2.1 Candidate point selection

First, *via* upsampling, the low-resolution segmentation map is converted to high-resolution, and N hard points with low confidence are filtrated in the high-resolution segmentation map. Most of these points are concentrated near the edge. This process iterates step by step to obtain a segmentation map of the desired resolution. In the implementation of this paper, the N value we choose is 28*28.

##### 2.2.2.2 Point feature extraction

Coarse and fine features for each candidate point are extracted. The coarse features are extracted from the low-resolution segmentation map, and the fine features are taken from the P2 layer of the FPN. The two sets of features are concatenated to obtain the feature expression of the candidate points.

##### 2.2.2.3 Point prediction

After obtaining the features of the candidate points, through a set of multi-layer perceptions (MLP), the final segmentation prediction results of the candidate points are obtained. More implementation details can be seen in Kirillov et al. (2020).

#### 2.2.3 Training and inference

##### 2.2.3.1 Training

In our implementation, we apply a multi-scale training mechanism (He et al. 2015). To address the issue of varying image sizes in training. In each epoch, a scale is randomly selected for training.

In the proposed pumpkin fruit and stem instance segmentation network, we define the training loss function as Equation (1):


(1) $$L=L_{classification}+\lambda L_{box}+\gamma L_{mask}$$

where *L_classification_* is the loss for fruit or stem classification, *L_box_* is the loss for the bounding box coordinates prediction, and *L_mask_* is the loss for mask prediction.

In our implementation, we apply cross entropy loss to calculate *L_classification_* and *L_mask_*, L1 loss to calculate *L_box_*. We set λ to 1 and γ to 2 because mask is more difficult to train and is more important in our implementation.

##### 2.2.3.2 Inference

The inference of the pumpkin fruit and stem instance segmentation network is a straightforward process. We forward input images through the transformer backbone and FPN. We select the points from the 224×224 resolution feature map refined by the coarse 7×7 prediction in 5 steps. We select the *N*=28^2^ most uncertain points based on the absolute difference between the predictions and 0.5.

### 2.3 Cutting and grasping point estimation

**Figure 8:**
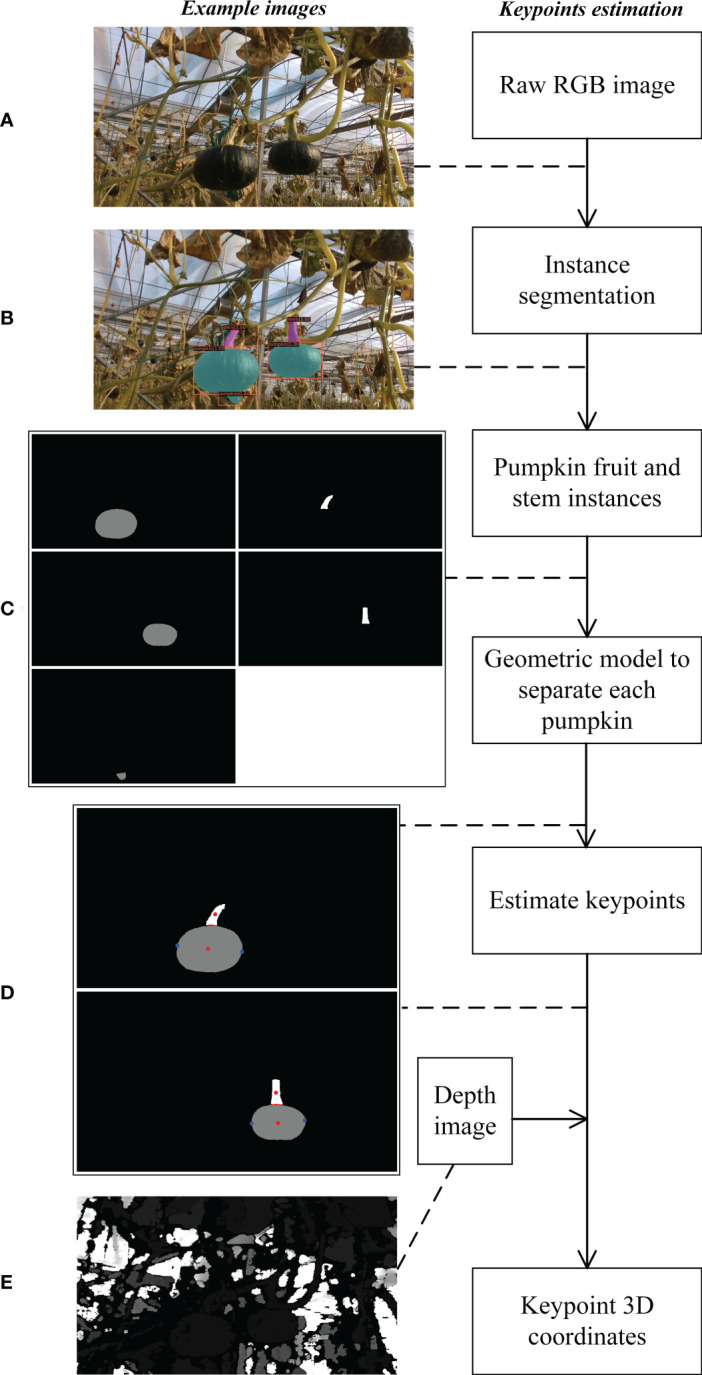
Block diagram of cutting and grasping point estimation method along with example images. **(A)** Pumpkin RGB image. **(B)** Pumpkin fruit and stem instance segmentation result. **(C)** Visualization of pumpkin fruit and stem instances. The left column instances are fruits, and the right column instances are stems. **(D)** The separate pumpkins with keypoints. The red points between the stem and the fruit are adjacent points. The red dot in the stem is the cutting point, and the blue points in the fruit are the grasping points. **(E)** Pumpkin depth image. Best viewed zoom in.

The proposed pumpkin keypoint detection framework is illustrated in **Figure 8**. Firstly, fruit and stem masks are generated *via* instance segmentation method as shown in **Figure 8B**. After obtaining the instance segmentation result, the fruit instances and the stem instances can be separated as depicted in **Figure 8C**. Among these instances, there are corresponding relationships among the fruits and the stems, and only one-to-one fruit and stem can be labeled as the pumpkin picking target. Then, we apply a geometric model to determine the cutting and grasping points. Finally, by modeling the robot and its coordinate systems, calibrating the camera parameters, the target pixel in 2D image can be transformed a position in 3D space. In practical operations, Birrell et al. (2020); Wang et al. (2022) and Kang et al. (2020) proposed approaches to tackle the coordinate transformation problem. Two pivotal steps of the keypoint estimation algorithm are fruit and stem correspondence determination and keypoint determination.

#### 2.3.1 Fruit and stem correspondence determination

To determine the corresponding relationships among the fruit and stem instances, we take advantage of the apriori knowledge. Three requirements should be satisfied. 1) The masks of fruit and stem are adjacent. 2) Under the force of gravity, the center point of the stem is above the center point of the fruit. 3) One fruit corresponds to at most one stem. **Algorithm 1** shows the matching process.

#### 2.3.2 Keypoint determination

After obtaining the mask of a whole pumpkin includes a fruit and a corresponding stem by the proposed correspondence determination algorithm, a geometric model is employed to estimate the exact location of the grasping points and cutting point.

**Figure 9:**
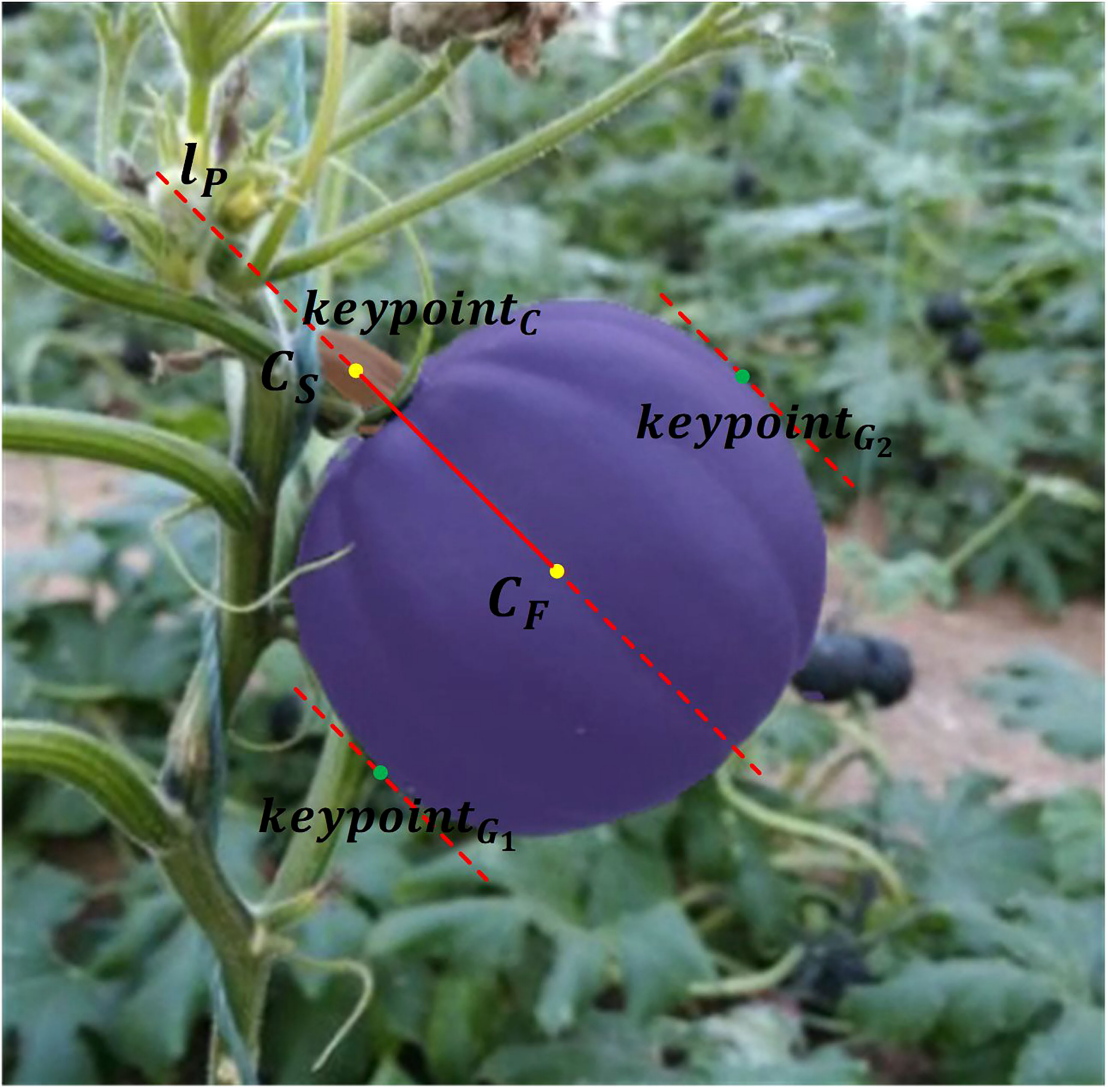
Estimation of pumpkin grasping and cutting points.


Denote fruit mask as $F:\;{\left\{\left(x_{i}^{F},y_{i}^{F}\right)\right\}}_{i=1}^{N_{F}}$ , stem mask as $S:\;{\left\{\left(x_{i}^{S},y_{i}^{S}\right)\right\}}_{i=1}^{N_{S}}$ , where *N_F_* and *N_S_* represents number of fruit pixels and stem pixels respectively. As illustrated in **Figure 9**, first, the center of mass of the 2D fruit and stem is calculated as Equation (2), labeled as $\left\{C_{F}:(x_{cf},y_{cf})\right\}$ and $\left\{C_{S}:(x_{cs},y_{cs})\right\}$ respectively.

(2) $$\begin{array}{c} x_{c_{f}}=\frac{\sum_{i=1}^{N_{F}}x_{i}^{F}}{N_{F}},\;y_{c_{f}}=\frac{\sum_{i=1}^{N_{F}}y_{i}^{F}}{N_{F}},\;x_{c_{s}}\\ =\frac{\sum_{i=1}^{N_{S}}x_{i}^{S}}{N_{S}},\;y_{c_{s}}=\frac{\sum_{i=1}^{N_{S}}y_{i}^{S}}{N_{S}} \end{array}$$

A straight line denoted as *l_p_* passes through *C_F_* and *C_S_*. Considering two conditions:

Algorithm 1 Matching pumpkin fruit masks with their corresponding stem masks.
**Input:**


$Fruit^{i}\in {\mathbb{N}}^{H\times W}$
: The *i*-th fruit mask in the image;


$Stem^{j}\in {\mathbb{N}}^{H\times W}$
: The *j*-th stem mask in the image;
*M*: Number of fruits detected in the image;
*N*: Number of stems detected in the image;
**Output:**
*K* matched pairs, each pair has a fruit mask and a stem mask, 

K≤min{M,N}
1: **for** i=0 to *M* **do**
2: Calculate center point of 
$Fruit^{i}:C_{F_{i}}=(x_{i},y_{i})$
3: **for** j=0 to *N* **do**
4: Calculate center point of 
$Stem^{j}:C_{S_{j}}=(x_{j},y_{j})$
5: **if** 
$x_{j}>x_{i}$
 (To ensure the center point of stem is above the center point of fruit) **then**
6: count adjacent mask point: 
num(dis<dis_thr)
7: **if** 
num(dis<dis_thr)>num_thr
 (To ensure the masks of fruit and stem are adjacent) **then**
8: 
$Fruit^{i}$
 and 
$Stem^{j}$
 is a matching candidate
9: **end if**
10: **end if**
11: **for** i=0 to *M* do
12: **if** There is one or more than one match candidates with stem **then**
13: Calculate the degree of pumpkin matching candidate: 
$\text{D=arctan}\frac{\left|x_{i}-x_{j}\right|}{\left|y_{i}-y_{j}\right|}$
14: The matching candidate with the minimum *D* value is determined as the match pair.(To ensure one fruit corresponds to at most one stem)
15: **end if**
16: **end for**
17: **end for**
18:**end for**

Case 1: *l_p_* is a vertical line (slope of *l_p_* is ∞).

Denote *l_p_* as *x*=*c*. Index of grasping points *G*_1_ and *G*_2_ from fruit mask *F* can be calculated as Equation (3):


(3) $$\{\begin{array}{l} G_{1}=\arg\;\underset{i}{\max}\;\left|x_{i}^{F}-c\right|,\;x\leq c\\ G_{2}=\arg\;\underset{i}{\max}\;\left|x_{i}^{F}-c\right|,\;x>c \end{array}$$

Case 2: *l_p_* is not a vertical line (slope of *l_p_* is not ∞).

Assume line equation as $l_{p}:y=kx+b$ . Denote *D_i_* as the distance from *i*th point in *F* to *l_p_*. *G*_1_ and *G*_2_ can be calculated as Equation (4):


(4) $$\left\{ \begin{array}{l} G_{1}=\arg\;\underset{i}{\max}\;D_{i},\;y_{i}^{F}\leq kx_{i}^{F}+b\\ G_{2}=\arg\;\underset{i}{\max}\;D_{i},\;y_{i}^{F}>kx_{i}^{F}+b \end{array}\right.$$

Finally, ${keypoint}_{G_{1}}:x_{G_{1}}^{F},y_{G_{1}}^{F}$ and ${keypoint}_{G_{2}}:x_{G_{2}}^{F},y_{G_{2}}^{F}$ are determined as two grasping points that distribute in two sides of *l_p_*. Cutting point ${keypoint}_{C}$ is the center point of stem mask, that is $\left\{C_{S}:(x_{c_{s}},y_{c_{s}})\right\}$ .

## 3 Results and discussion

### 3.1 Evaluation metrics

#### 3.1.1 Average precision

According to whether the true sample and the predicted result match, the prediction results can be divided into four types: true positive (TP), false positive (FP), true negative (TN), and false negative (FN). Precision and recall are defined as follows:


$$Precision=\frac{TP}{\left(TP+FP\right)}$$

$$Recall=\frac{TP}{\left(TP+FN\right)}$$

The average precision metric is used to measure the quality of the detections and the segmentations obtained by the models. Average precision computes the average precision value for recall values over 0 to 1. Specifically, mean average precision (mAP) is defined as the primary metric. As in (Lin et al. (2014)), mAP is calculated using 10 intersection over union (IoU) thresholds from 0.50 to 0.95. The IoU measures the overlap between two boundaries or masks and measures how much the box boundary or mask predicted by the algorithm overlaps with the ground truth (the real object boundary or real object mask).

#### 3.1.2 Model complexity and inference speed

The model complexity usually relates to parameter number and calculation amount, two metrics that describe how many parameters the model defines and how many floating point operations(FLOPs) are required when running the model. 1GFLOPs = 10^9^FLOPs. The metric to define the model inference speed is the average number of frames per second (FPS). Model complexity and FPS are vital indicators to evaluate the performance of the model.

### 3.2 Instance segmentation result

#### 3.2.1 Experiment setup

In this paper, the training and evaluation of the proposed network are conducted on a server, which consists of an Intel i9-10900X CPU with 20 cores, 32G RAM, and an RTX 3090 GPU with 24G memory. The network implementation was carried out using Pytorch 1.7.0.

#### 3.2.2 Performance comparison with state-of-the-art methods

**Figure 10:**
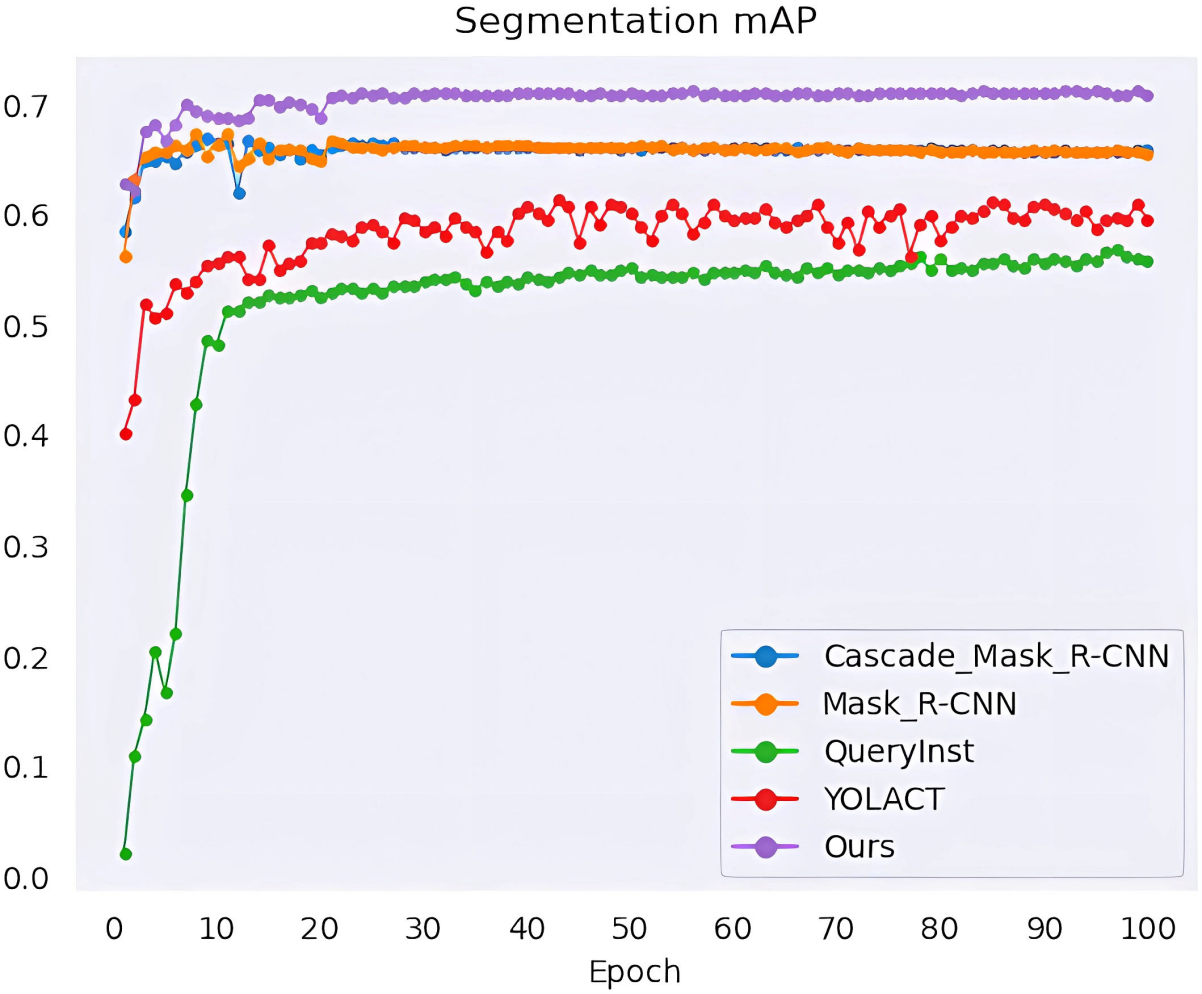
Mask segmentation mAP of the model.

**Figure 11:**
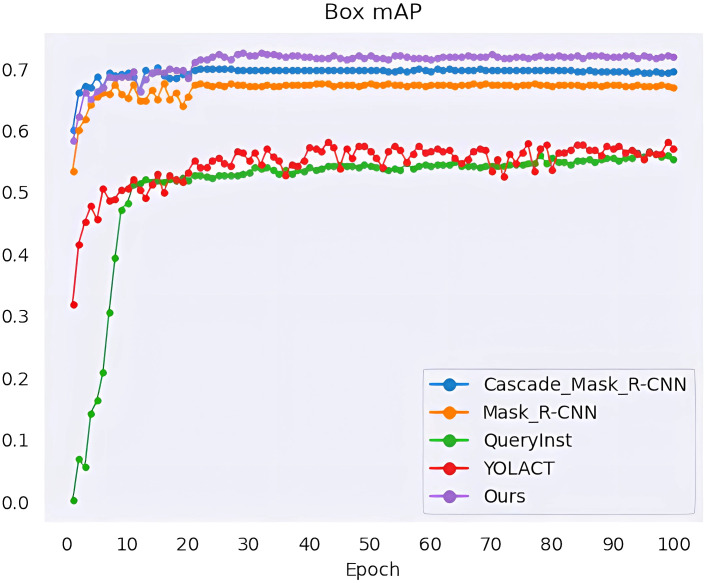
Box detection mAP of the model.

We performed a series of experiments to compare our method with the state-of-the-art methods, namely YOLACT (Bolya et al., (2019), QueryInst (Fang et al., 2021), Mask R-CNN (He et al., (2017) and Cascade Mask R-CNN (Cai and Vasconcelos, 2019). All algorithms are trained for 100 epochs, and when every training epoch ends, the mAP values of mask segmentation and box detection are calculated as shown in **Figures 10**, **11**. The detection mAP of our proposed method outperforms these state-of-the-art methods, and the segmentation mAP is significantly superior to the existing methods. Fortunately, in this application, segmentation precision is more important than detection precision.

The evaluation results are listed in **Table 2**. Our architecture achieves a high instance segmentation accuracy of 0.708 mask mAP and 0.720 box mAP, which brings 4.9% and 2.5% gains over the second-best results. From the parameter comparison, except QueryInst (the model is too large) and YOLACT (the accuracy is not satisfactory), the margin among parameter numbers of Mask R-CNN, Cascade Mask R-CNN and the method we proposed is narrow. It’s worth noting that although the parameter size of our method is larger than Mask R-CNN (59.27M Vs 43.76M), the computational complexity is lower than Mask R-CNN (213.01 GFLOPs Vs 258.19 GFLOPs). Our method achieves 13.5 FPS on a single RTX 3090 GPU, which can meet the requirements of agricultural applications.

**Table 2:** Performance comparison with state-of-the-art methods.

	mask mAP	box mAP	#param.	GFLOPs	FPS
YOLACT (Bolya et al. (2019))	0.596	0.572	**34.74M**	**186.57**	**21.3**
QueryInst (Fang et al. (2021))	0.559	0.554	172.23M	464.29	6.2
Mask R-CNN (He et al. (2017))	0.656	0.669	43.76M	258.19	16.4
Cascade Mask R-CNN (Cai and Vasconcelos (2019))	0.659	0.695	76.8M	389.03	13.6
Ours	**0.708**	**0.720**	59.27M	213.01	13.5

The best performances of each metrics are in bold format.

#### 3.2.3 Visualization result analysis

**Figure 12:**
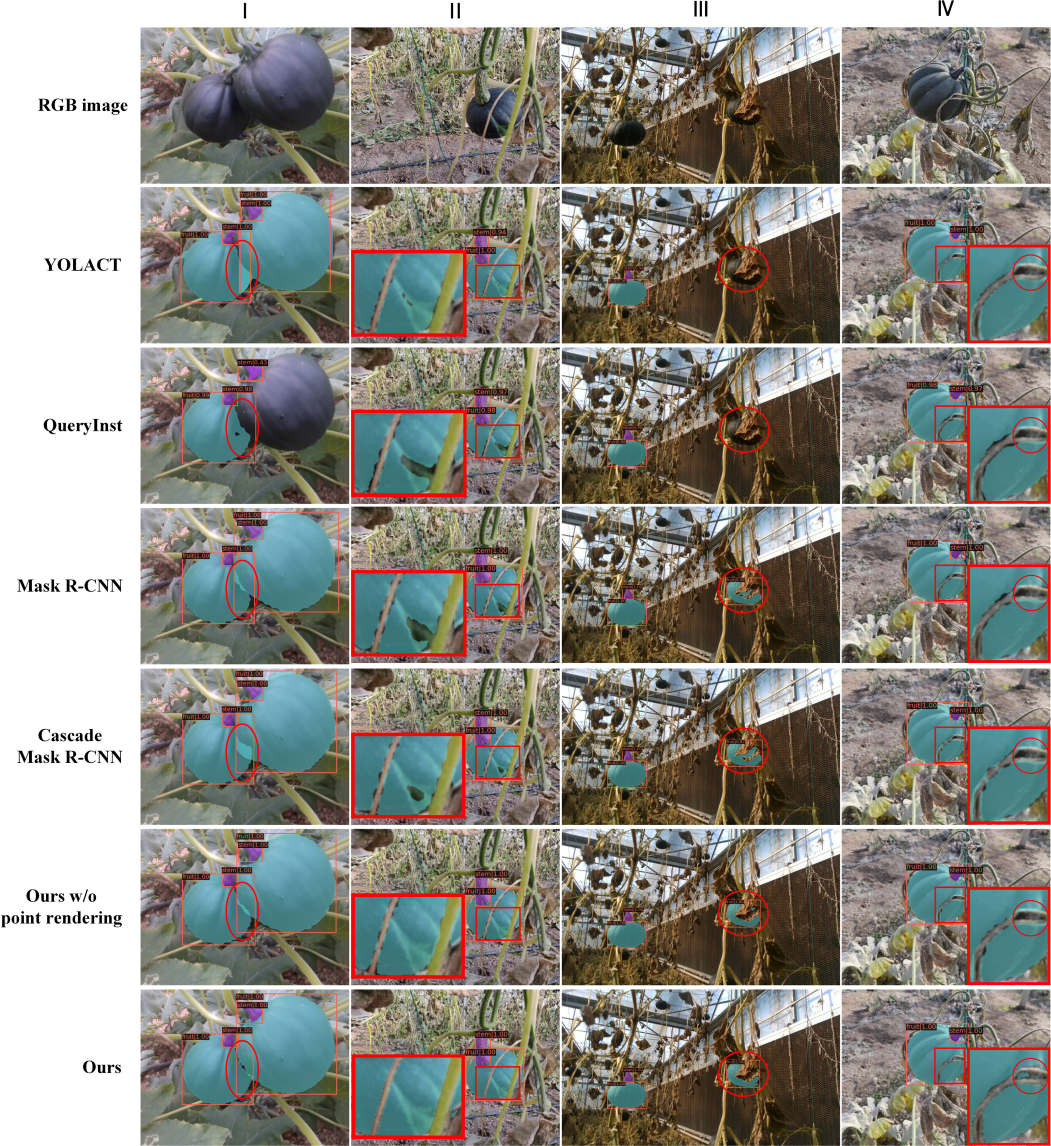
Example of pumpkin fruit and stem instance segmentation results.

To highlight the superiority of the proposed architecture more intuitively, the visual analysis of the outstanding networks and our network is conducted. As can be seen in **Figure 12**, all methods can detect the majority of pumpkin instances, whereas our method achieves higher confidence. As is shown in the third column, YOLACT and QueryInst fail to detect the pumpkin in red circle covered by the leave, while Mask R-CNN, Cascade Mask R-CNN and our method detect the pumpkin with the confidence of 0.38, 0.97 and 1.0, respectively. It is obvious that our method generates finer masks compared with other methods. To emphasize the contribution of point rendering mask branch, we compared the visualization results of our method and our method without point rendering as shown in the last two rows, where can be seen that the finer masks benefit more from the point rendering mechanism.

#### 3.2.4 Ablation study of improved models

In order to prove the effectiveness of the improved modules in the proposed pumpkin fruit and stem instance segmentation method, the ablation study on different modules is performed in this section. The comparisons are conducted on seven cases, as shown in **Table 3**. As can be seen from the table, replacing the original CNN module with the transformer network and the mechanism of multi-scale training have greatly improved the results, and the mask mAP and box mAP have increased by 2.5%, 2.4% and 1.7%, 3.3% respectively. Although the improvement of replacing the original mask branch with the point rendering mask branch takes no remarkable superiority in mAP results, it only increases by 0.6% in mask mAP, and the box mAP has a slight increase of 0.3%, but from the visualization results, point rendering mask branch greatly optimizes the boundary masks, which cannot be ignored. Finally, the architecture with transformer network, point rendering mask branch, and the multi-scale training network improves 5.3% mask mAP and 5.1% box mAP over the Mask R-CNN Baseline network. The inference speed decreased from 16.4 FPS to 13.5 FPS, but this is acceptable.

**Table 3:** Ablation study on the pumpkin fruit and stem instance segmentation method.

Model	transformer network	multi-scale training	point rendering mask	mask mAP	box mAP	FPS
Baseline model				0.656	0.669	16.4
Model-A	√			0.681	0.686	15.4
Model-B		√		0.680	0.702	**16.5**
Model-C			√	0.665	0.672	14.3
Model-D	√	√		0.705	0.718	15.1
Model-E		√	√	0.701	0.709	13.7
Model-F (Ours)	√	√	√	**0.708**	**0.720**	13.5

The best performances of each metrics are in bold format.

### 3.3 Keypoint estimation results

#### 3.3.1 Pumpkin fruit and stem correspondence determination result

**Figure 13:**
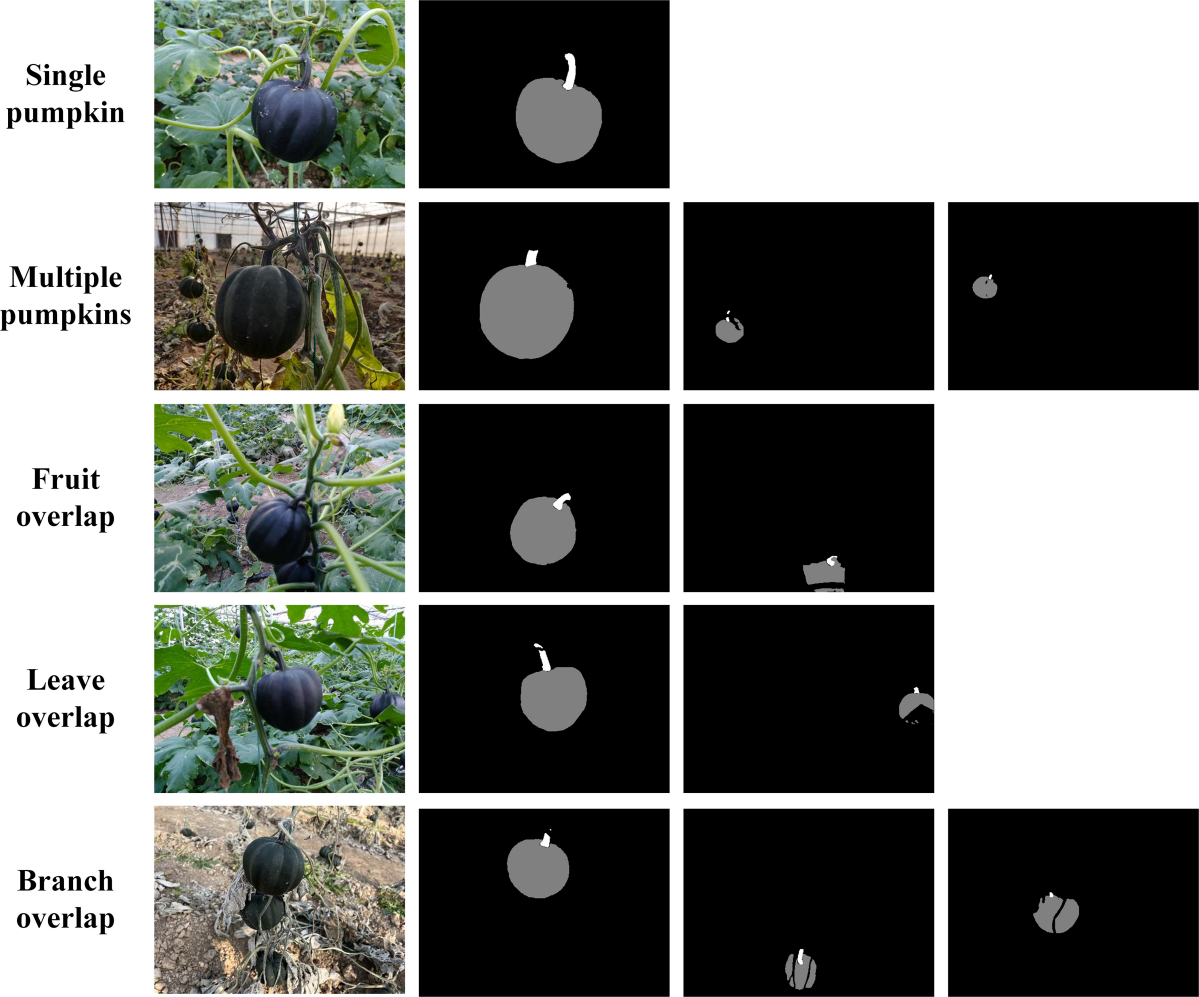
Pumpkin fruit and stem matching result.

**Figure 14:**
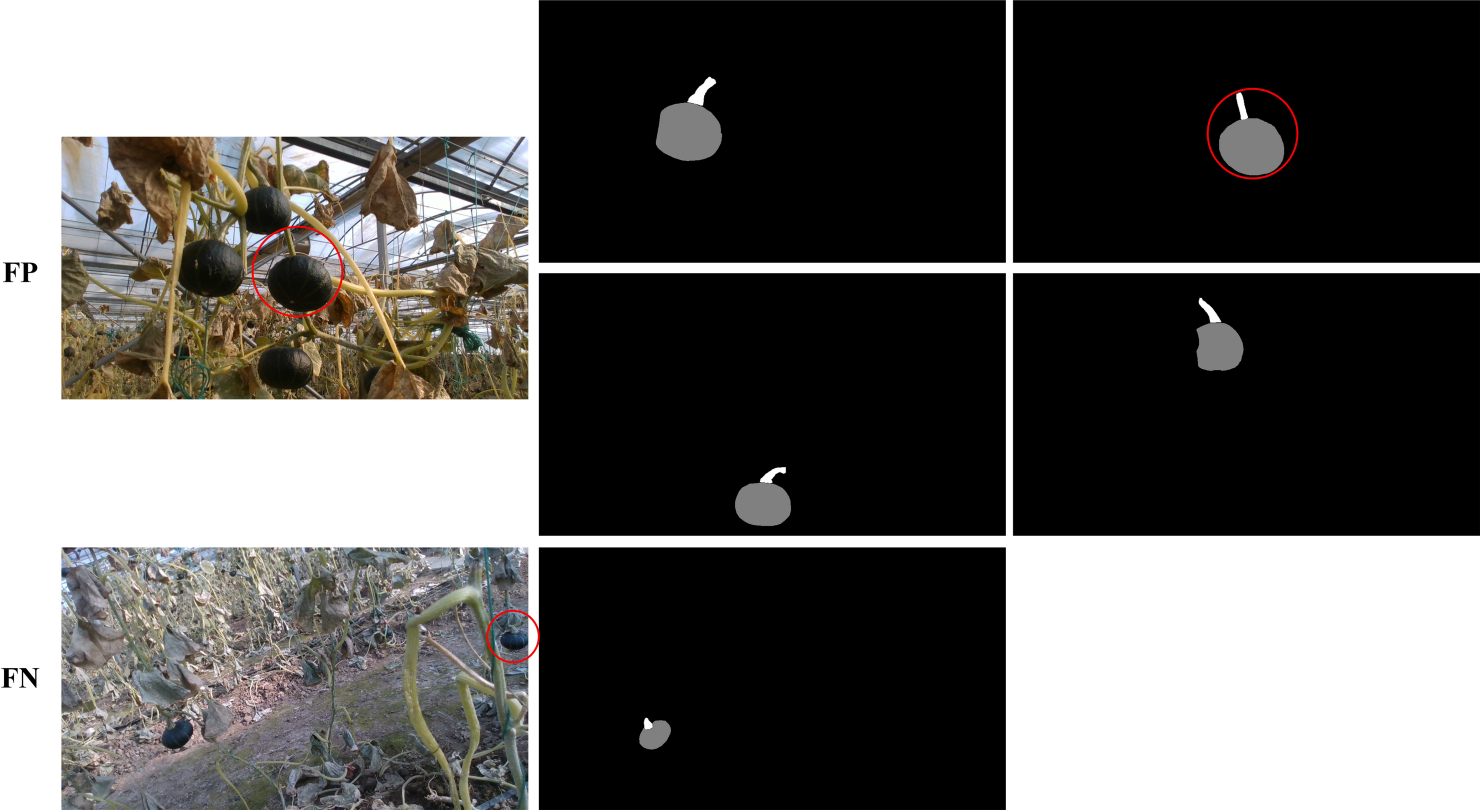
Wrong examples of pumpkin fruit and stem matching result.

**Figure 13** shows some example results of fruit and stem matching algorithm. It can be seen that in most conditions, including one image with single or multiple pumpkins, existing fruit, leave, or branch overlaps, our algorithm can match the fruits and stems successfully. To analyze the results accurately, we count all the matched pumpkin instances in the test images, the number of TP is 215, FP is 4, and TN is 2. The precision and recall reach 98.2% and 99.1% respectively. Some negative matched examples are listed in **Figure 14**. The reason for the faults is that in the instance segmentation step, missing and erroneous detections happen sometimes. The pumpkin is too small or interference of branches may cause false detection.

#### 3.3.2 Keypoint determination result

**Figure 15:**
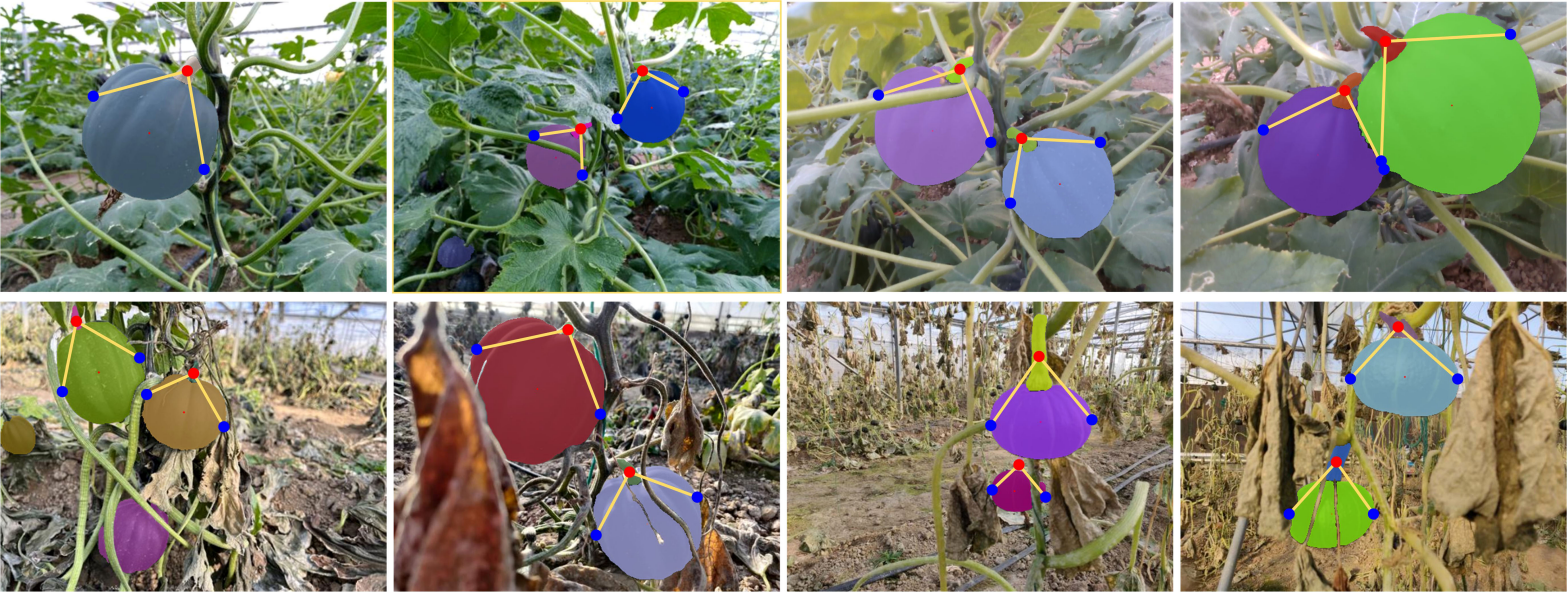
Visualized results of grasping and cutting keypoint detection.

**Figure 15** presents the visualized results of grasping and cutting keypoint detection. In the figure, red points are the cutting points, blue points are the grasping points. Yellow lines linking the cutting points and grasping points signify that the 3 points attach to one pumpkin instance. Specially, the occlusion problem is usually not negligible in fruit picking task. One of the advantages of this approach is that if a pumpkin is occluded seriously, for instance, if we can only see the fruit part or the stem part in the image, our algorithm can filter this pumpkin autonomous as shown in the first image from the second row in **Figure 15**. If the pumpkin is only occluded part of the fruit or stem by leaves, branched or other fruits, our algorithm also determines the grasping point and cutting point reasonably as shown in the right three columns from **Figure 15**. The results show that our algorithm is promising for the pumpkin picking task.

## 4 Conclusion

In this paper, we presented a pumpkin autonomous picking framework with keypoint detection and instance segmentation method. A transformer network is utilized as the architecture backbone to replace CNN, which helps achieve a higher detection and segmentation precision. To tackle the overlapping problem, point rendering is applied so that finer masks can be acquired. Sufficient experimental results indicate that our method significantly outperforms several state-of-the-art instance segmentation methods. In addition, a novel keypoint detection algorithm is proposed to model the relationships among the fruit and stem instances as well as estimate grasping and cutting keypoints. The effectiveness and applicability of the proposed method are verified through plenty experiments on pumpkin image dataset we created. In this work, we applied traditional geometric method to model the fruit-stem relationships and estimating the keypoints. Our future work will expand into learning-based method to detect the fruit-stem pairs and directly generate the keypoints using deep neural networks.

## Data availability statement

The original contributions presented in the study are included in the article/supplementary material. Further inquiries can be directed to the corresponding author.

## Author contributions

JY, YL, DZ, and TX participated in the conception and design of this research and revised the manuscript. JY carried out the experiments, organized the database, performed data analysis, and wrote the manuscript. YL, DZ, and TX advised on the design of the model and analyzed to find the best method for this work. All authors contributed to the article and approved the submitted version.
